# CD99: A Cell Surface Protein with an Oncojanus Role in Tumors

**DOI:** 10.3390/genes9030159

**Published:** 2018-03-13

**Authors:** Maria Cristina Manara, Michela Pasello, Katia Scotlandi

**Affiliations:** Laboratory of Experimental Oncology, CRS Development of Biomolecular Therapies, Rizzoli Orthopaedic Institute, 40136 Bologna, Italy; michela.pasello@ior.it

**Keywords:** CD99, tumor progression, antibodies, targeted therapies

## Abstract

The cell surface molecule CD99 has gained interest because of its involvement in regulating cell differentiation and adhesion/migration of immune and tumor cells. However, the molecule plays an intriguing and dual role in different cell types. In particular, it acts as a requirement for cell malignancy or as an oncosuppressor in tumors. In addition, the gene encodes for two different isoforms, which also act in opposition inside the same cell. This review highlights key studies focusing on the dual role of CD99 and its isoforms and discusses major critical issues, challenges, and strategies for overcoming those challenges. The review specifically underscores the properties that make the molecule an attractive therapeutic target and identifies new relationships and areas of study that may be exploited. The elucidation of the spatial and temporal control of the expression of CD99 in normal and tumor cells is required to obtain a full appreciation of this molecule and its signaling.

## 1. Introduction

**Figure 1:**
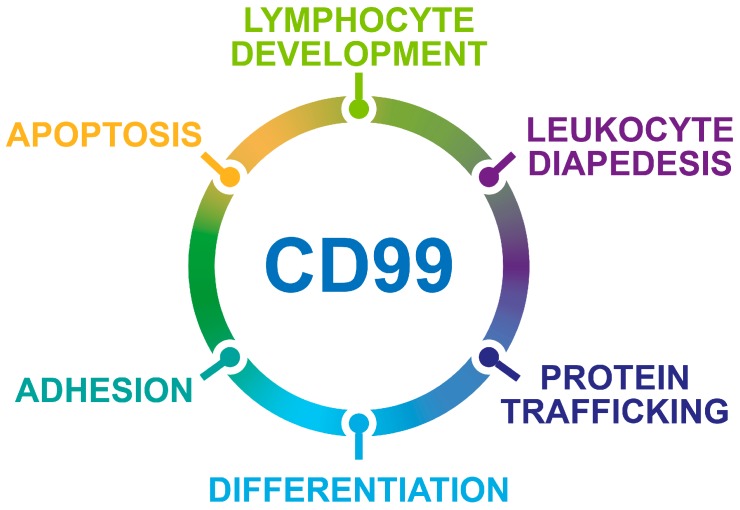
Schematic representation of the biological processes regulated by CD99.

CD99, a glycosylated transmembrane protein, is involved in many essential cellular functions. For example, cell adhesion and migration, cell death and differentiation, intracellular protein trafficking, endocytosis and exocytosis are influenced by the CD99-regulated signaling. Figure 1 schematizes the effects of CD99 on biological processes. In tumors, CD99 has been reported to have a marked effect on the migration, invasion, and metastasis of tumor cells through multiple and still controversial mechanisms of action, thereby emerging as a novel therapeutic target. However, much still needs to be understood. High CD99 expression has been observed in Ewing sarcoma [1,2], lymphoblastic lymphoma/leukemia [3], myeloid malignancies [4], malignant glioma [5,6], and sporadically, in synovial sarcoma [7], mesenchymal chondrosarcoma [8], rhabdomyosarcoma [9], thymic tumors, hemangiopericytoma [10], gastrointestinal and pulmonary neuroendocrine tumors [11], sex-cord stromal tumors [12], melanoma [13], and a small percentage of breast carcinomas [14]. In all these tumors, CD99 has been reported to exert oncogenetic functions. However, there is an emerging group of neoplasms, including pancreatic endocrine neoplasms, gastric adenocarcinoma [15,16], gallbladder carcinoma [17], and osteosarcoma [18], in which CD99 expression is diffuse in benign or early-stage tumors and absent or lower in the malignant/advanced-stage counterparts. In this set of tumors, CD99 delivers oncosuppressor signaling, and its re-expression leads to reversal of malignancy. In addition, the *CD99* gene encodes two distinct proteins as a result of the alternative splicing process of the cytoplasmic region: a wild-type full-length CD99, or CD99 type I (CD99wt), containing 185 aminoacids (corresponding to a molecular weight of 32 kDa), and a truncated form, or CD99 type II (CD99sh), containing 161 aminoacids (corresponding to a molecular weight of 28 kDa) [19]. The CD99 isoforms are expressed in a cell-type-specific manner and dictate distinct, dualistic CD99 functions [20,21,22]. This finding adds another level of complexity to our understanding of CD99 mechanisms of action. This review highlights key examples of the opposite roles of CD99 in tumor progression and identifies new questions that need to be addressed to conveniently exploit the therapeutic potential of a molecule that has a relevant impact on tumor biology but has been largely ignored by the scientific community.

**Figure 2:**
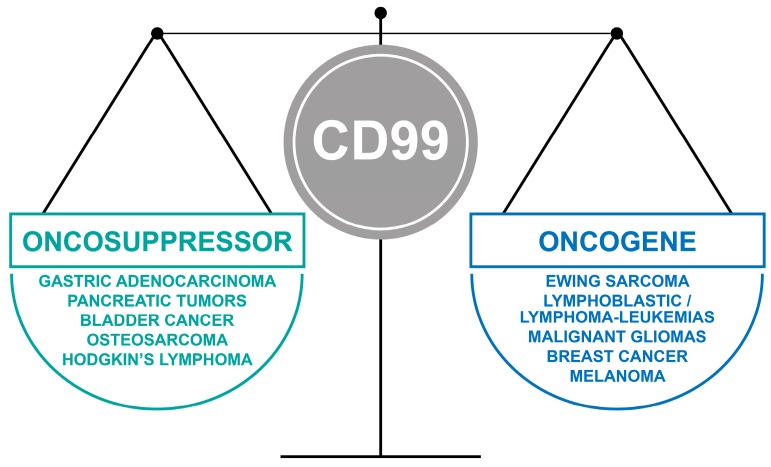
The oncojanus function of CD99 in tumors.

Key points:

- The opposite, dual functions of CD99 isoforms.
- The oncojanus function of CD99 in tumors **(**Figure 2).
- The therapeutic potential of strategies targeting CD99.
- Critical issues and novel perspectives.

## 2. CD99 Structure and Expression in Normal Tissues

CD99 is encoded by the *MIC2* gene [23], which is located in the human pseudoautosomal region in the distal short arms of the X and Y chromosomes [24]. The molecule is highly *O*-glycosylated, and CD99, Xga, and CD99L2 constitute a family of molecules with no homology to any other known family [25,26,27,28]. The mRNA of CD99 is expressed in two isoforms, a wild-type full-length CD99 (CD99wt, also known as CD99 type I) of 185 amino acids and a short form of 161 amino acids (CD99sh, also known as CD99 type II) obtained by alternative splicing of the cytoplasmic region: this splicing introduces an in-frame stop codon that generates a truncated polypeptide [19]. The CD99wt protein has a molecular weight of 32 kDa and is formed by an extracellular domain, which is glycosylated with O-linked sugar residues, followed by a transmembrane domain and a 36-amino acid intracytoplasmic domain [29]. The CD99sh protein has a molecular weight of 28 kDa and displays a deletion in its intracytoplasmic fragment. The two isoforms of CD99 can naturally dimerize on the cell surfaces.

The isoforms of CD99 transcripts are differentially expressed in a cell type-specific manner among hematopoietic cells, with CD99wt predominantly expressed [19,30]. Single-positive thymocytes and peripheral T cells display an exclusive expression of the full-length CD99wt isoform, whereas double-positive thymocytes and some immature T cell lines express both isoforms [20], indicating a tight correlation with T cell differentiation. In addition, the expression of the CD99 isoforms correlates with that of CD1a in human thymocytes; double-positive thymocytes express CD1a and the two forms of CD99, while single-positive thymocytes that have lost the expression of CD1a express only the long form of CD99 [31]. Among B cells, B cell precursors with acute lymphoblastic leukemia (BCPs-ALL) express mainly the long form of CD99, while in normal B cell precursors (BCPs), CD99 is downregulated during differentiation. In contrast, CD99sh is barely expressed in both normal BCPs and in BCPs-ALL with no effect on maturation [32].

However, few studies have specifically considered how the two isoforms are expressed in appropriate cellular contexts and how they affect CD99-mediated intracellular pathways. In most cases, evidence is derived from studies using antibodies directed against the extracellular domain of CD99 and, therefore, incapable of discriminating between CD99wt and CD99sh. Therefore, except when specifically noted, this review refers mostly to data on human CD99wt.

In normal tissues, the expression level of CD99 is particularly high in cortical thymocytes, pancreatic islet cells, granulosa cells of the ovary, and Sertoli cells of the testis. In addition, CD99 has been found to be expressed at high levels in CD34^+^ cells of the bone marrow and in all leukocyte lineages, with the highest expression in the most immature lymphocytes and granulocytes (for a review see [33]). CD99 has been found to participate in various steps of T and B cell development and differentiation [30,34,35] and it regulates crucial functions, such as the proliferation and activation of T lymphocytes [36,37], apoptosis [30,38], adhesion [19,34,39,40], lymphocytes diapedesis to the inflamed vascular endothelium [41,42], and intracellular membrane protein trafficking [43,44,45,46]. In particular, CD99 plays an active role in the transport and expression of major histocompatibility complex (MHC) class I and II and in TCR expression on thymocytes [43,44,45] and in the regulation of CD1a expression on dendritic cells [31]: thus, CD99 is a critical determinant in the orientation of the immune response.

Strong expression of CD99 has also been reported in immature basal keratinocytes [47], in osteoblasts [18,48], and, at variable levels, in mesenchymal stem cells [49]. For more details refer to Pasello [33].

## 3. CD99 Isoforms Dictate Opposite Functions in Physiology and Malignancy

The differential expression levels and functions of alternative splicing products are among the physiological and pathological features of many cell adhesion molecules. Regarding CD99, several studies have shown that, by encoding for CD99wt and/or CD99sh, the *MIC2* gene can positively or negatively regulate cellular adhesion, apoptosis, migration, and metastasis. Indeed, CD99sh frequently plays a negative role in CD99wt-dependent functions, counteracting its effects [21,22]. It has been postulated that truncation of the cytoplasmic domain of the CD99 short form alters the three-dimensional structure of the molecule [50], leading to different binding sites for its ligand. However, the overall scenario is far from being understood, and only sporadic and controversial information is available. In B cells, the minor splicing form of CD99 was shown to inhibit homotypic adhesion, while the activation of the long CD99wt form promoted cell–cell adhesion via the opposite regulation of the expression of the cell adhesion molecule LFA-1/ICAM1 [19]. In contrast, no effect of CD99 on the αLβ2 integrin/ICAM1 pathway has been reported within T cells [20]. Transfection into CD99-deficient Jurkat T cells of both CD99wt and CD99sh was required to induce apoptosis via CD99, whereas transfection of a single chain (either wt or sh) could induce apoptosis but was sufficient to modulate adhesion events triggered via CD99 [20]. When co-expressed, the two isoforms form covalently bound heterodimers that localize within glycosphingolipid rafts and induce sphingomyelin degradation. Cholesterol depletion experiments have shown that this localization is required for the induction of apoptosis [20]. However, this is not a general observation. In Ewing sarcoma, which prevalently expresses CD99wt, cells are prone to die after CD99 engagement by specific antibodies via (see below) non-apoptotic mechanisms [51,52,53]. This finding further supports the need for additional studies to elucidate the relationship between the two isoforms and their effect on cellular signaling. CD99 isoforms have also been found to have counteracting effects on the regulation of the expression of CD1, a non-classical MHC molecules that ensures the presentation of lipid and glycolipid antigens, during dendritic cells differentiation [31], whereas MHC class I and II are associated with both CD99 isoforms [54].

**Figure 3:**
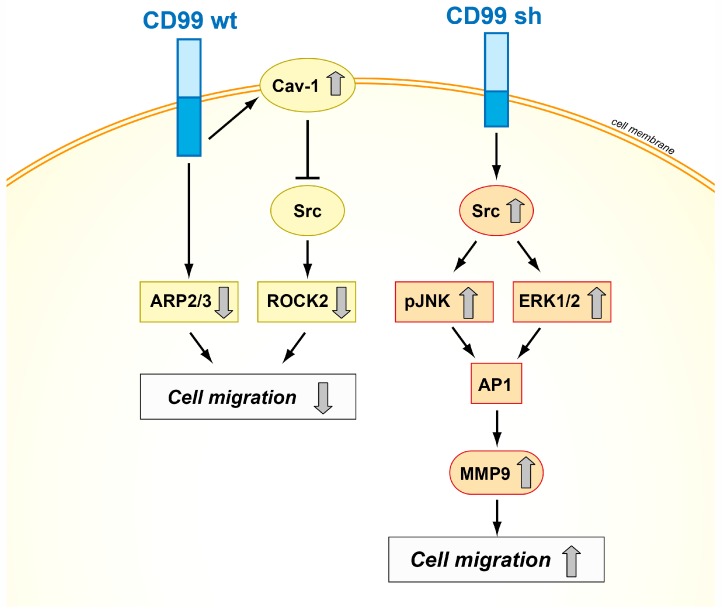
Schematic representation of the mechanisms of action of CD99wt and CD99sh in tumor cells. The two isoforms exhibit opposite effects on cell migration, inhibiting or activating the Src signaling pathway [18,21,53].

CD99 isoforms also dictate different functions in malignancy, exerting opposite effects on crucial biological processes, such as migration/invasion, growth in anchorage-independent conditions, and differentiation and metastasis in osteosarcoma, prostate cancer, and breast carcinoma [21,22,55]. From a mechanistic point of view, several signal transducing molecules, including MAPKs, Src kinase, and protein kinase C (PKC) have been found to mediate CD99-dependent processes [40,56]. It has been demonstrated that the cytoplasmic domain of the long form contains two putative phosphorylation sites, a serine at amino acid residue 168 and a threonine at amino acid residue 181. These two residues may be important for intracellular signaling events and/or extracellular molecular interactions [22]. The Ser168 of CD99 long form has been reported to be a site for PKCα phosphorylation [20], which induces focal adhesion kinase (FAK) phosphorylation and is thereby, in turn, involved in actin polymerization and cell adhesion and migration [57]. However, no information is available on the intracellular signaling that may be associated with Ser168 phosphorylation. Up to now, data available in osteosarcoma, only pointed out the importance of the presence of the Ser168 residue of CD99: Ser168 is required for the CD99wt-mediated inhibition of migration and metastatization of osteosarcoma and prostate cancer cells [22]. Ser168 is not present in CD99sh, consistent with the positive contribution of CD99sh to tumor malignancy. The expression of CD99sh in Ewing sarcoma inhibited cell differentiation and contributed to maintenance of stemness [58,59]. In human breast cancer, expression of the splice variant of CD99 increased the activity and expression of MMP-9 and contributed to the invasive phenotype by upregulating AP1-mediated gene expression through the AKT-dependent, ERK, and JNK signaling pathways [21]. In addition, the two isoforms can either activate or repress c-Src family kinase activity, thereby providing at least a conceptual basis for their dualistic functions in modulating cell migration, anchorage-independent growth, and metastasis (Figure 3) [22,60].

However, with its short cytoplasmic tail, it is unlikely that CD99 itself takes part in signaling events. The functions of CD99 may depend on its association with other membrane proteins, possibly explaining the dual roles of CD99 and its isoforms in various cell types.

## 4. When CD99 Is Associated with Malignancy and Highly Expressed

**Figure 4:**
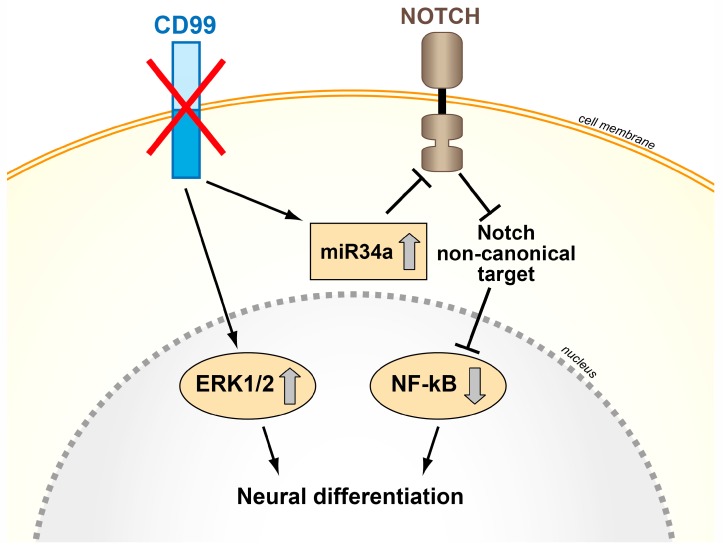
Schematic representation of the effects of CD99 silencing in Ewing sarcoma cells. The abrogation of CD99 induces Ewing sarcoma neural differentiation through nuclear ERK1/2 stabilization and miR34a upregulation and Notch-NF-kB inhibition [59,74].

High expression of CD99 is a distinctive feature of Ewing sarcoma. Moreover, the detection of CD99 is routinely used for the differential diagnosis of conventional Ewing sarcoma with respect to other types of small round-cell tumors of childhood, including lymphomas, small round-cell osteosarcoma, and the most recently defined Ewing-like round cell tumors, a group of very rare malignancies morphologically resembling Ewing sarcoma but carrying a different type of genetic alterations and exhibiting lower and dispersed expression of CD99 [1,2,61,62,63]. The consistent, high-level expression of CD99 and the presence of a *EWS* gene-rearrangement with *FLI1*, *ERG* or, in rare cases, other *ETS* genes [64,65] are the hallmarks of Ewing sarcoma, and a functional relation exists between the two, although that relation remains unclear. The introduction of EWS-FLI1 into neuroblastoma, rhabdomyosarcoma, or mesenchymal stem cells turned on CD99 expression [66,67] while the silencing of EWS-FLI1 in Ewing sarcoma cells does not affect high-level CD99 expression [68]. The prevailing idea is that the oncogenic potential of EWS-FLI1 is facilitated by the presence of CD99: however, CD99 actively participates in the maintenance of tumor malignancy. In fact, Ewing sarcoma cells deprived of CD99 but still presenting EWS-FLI1 showed dramatically inhibited growth, migration, and metastatic capabilities and were prone to differentiate toward the neural lineage [59,69]. These results imply that constitutive CD99 expression supports Ewing sarcoma cell malignancy. Accordingly, EWS-FLI1 maintains or induces the expression of the molecules either directly through its binding to *CD99* promoter [49,59] or indirectly through miRNA regulation [68]. From a mechanistic point of view, the results obtained to date suggest that high CD99 expression levels may contribute to tumor cell growth and dissemination by interfering with the following: (1) the migration inhibitory activity of KCMF1 [69], a potassium channel modulatory factor reported to affect the biological function of 14-3-3σ protein and stabilize MAPK signaling [70,71], and (2) PI3K/AKT signaling, which is generally repressed, and MAPK signaling, which is stabilized in CD99-deprived cells or after CD99 ligation with specific antibodies [21,56,59]. Particularly relevant and consistent is the sustained activation of the RAS/MAPK pathway in Ewing sarcoma cells deprived of CD99. This pathway is conventionally associated with cell proliferation and, in certain primary vertebrate cells, including neuronal, adipocytic, and myeloid cells, can induce differentiation, in some cases accompanied by growth arrest (for a review see [72]). This function may be related to signal duration: a transient pathway activation was shown to result in cellular proliferation, whereas a prolonged pathway activation resulted in neural differentiation [73]. Therefore, CD99 loss induced in Ewing sarcoma cells prolongs nuclear ERK1/2 phosphorylation, which appears to be crucial for shifting the biological functions of ERK1/2 toward neural development and differentiation. In addition to the induction of ERK1/2, the silencing of CD99 was shown to lead to increased expression of the oncosuppressor miR34a, which in turn could both reduce both Notch 1 and NF-κB signaling [74]. Notably, the same outcome was obtained by exposing Ewing sarcoma cells to exosomes derived from cells deprived of CD99 [74], confirming the sequential path of miR34a/Notch/NF-κB and MAPK upregulation that follows CD99 silencing. Figure 4 schematizes the CD99-related signaling in Ewing sarcoma cells. Overall, CD99 is required for the Ewing sarcoma oncogenic phenotype. The molecule appears to function by blocking differentiation while supporting growth and cell migration.

Similar evidence has been obtained in hematopoietic tumors. CD99 is highly expressed in T-lineage acute lymphoblastic leukemia (T-ALL) [3], early B cell lymphoblastic lymphomas [35], immunophenotypic acute myeloid leukemia (AML) [4,75,76], and myelodysplastic syndromes (MDS) stem cells [4]. CD99 is frequently expressed in disease-initiating stem cells in MDS and AML, and its detection could allow the prospective separation of leukemic stem cells from functionally normal hematopoietic stem cells in AML, thereby demonstrating the potential of anti-CD99 monoclonal antibodies as therapeutic agents.

The inverse relation between CD99 expression and differentiation has also been confirmed in thymic tumors, in which CD99 is useful for identifying immature T cells and for distinguishing thymoma from thymic and non-thymic carcinomas [77].

CD99 is also upregulated in tumors of the nervous system. CD99 was found to be highly expressed in ependymomas [78,79], in malignant gliomas [5], and in astrocytomas [5,6], where its detection can help discriminate among tumor grades, with a differential subcellular distribution according to the malignancy stage. Knocking down CD99 expression by siRNA in cell lines derived from these tumors significantly decreased cell migration, further suggesting that CD99 may contribute to the infiltrative ability of tumor cells. Interestingly, Rac activity was decreased and Rho activity was increased in CD99-overexpressing glioma cells [5], together with the ratio of amoeboid cells to mesenchymal cells.

Taken together, these findings suggest that CD99 may play an important role in the migration and invasion of certain human tumors and significantly affect crucial signaling pathways, such as AKT, ERK, and Rho/Rac signaling pathways. However, even if the final effect of CD99 overexpression is the same in all these tumors, the intracellular mechanisms of action are far from being commonly defined and strictly cellular context-dependent.

## 5. When CD99 Acts as an Oncosuppressor and is Lost in Malignancy

In Hodgkin’s lymphomas [80], osteosarcomas [18,22], pancreatic tumors [11], gallbladder carcinomas, gastric carcinomas [15,17] and in pulmonary neuroendocrine tumors of diverse histological types [81], CD99 is expressed at low levels, whereas the molecule is present in the respective normal tissues. In gastric adenocarcinoma samples [82] and in pulmonary carcinoid tumors [83], the decreased expression of CD99 has clinical significance, as it is strongly associated with poor survival and a heightened risk of metastasis formation. Notably, the forced expression of CD99 in osteosarcoma and/or in stomach cancer cell lines has been observed to reduce proliferation, migration, and metastasis capabilities [18,22,55,82], while increasing cell differentiation [48]. It has also been shown that CD99 downregulation leads to the loss of normal morphology in Hodgkin’s disease [80], while the upregulation of CD99 in Hodgkin/Reed–Sternberg cells induces terminal B cell differentiation [84]. Therefore, in all these tumors, the downregulation of CD99 protein expression is a critical event in tumor progression and is related to the loss of cellular identity or differentiation and of intercellular adhesion capability with increased migratory/invasion cell capabilities.

Methylation of the CD99 promoter, CD99 loss of heterozygosity (LOH), and loss of SP1 expression, which is a potent positive regulator of CD99, can account for CD99 downregulation in most cases [82,85]. However, only sporadic studies are available. The involvement of CD99 mutations, posttranslational modifications, such as regulation by miRNAs, and additional derangements of other transcription factors may also occur.

**Figure 5:**
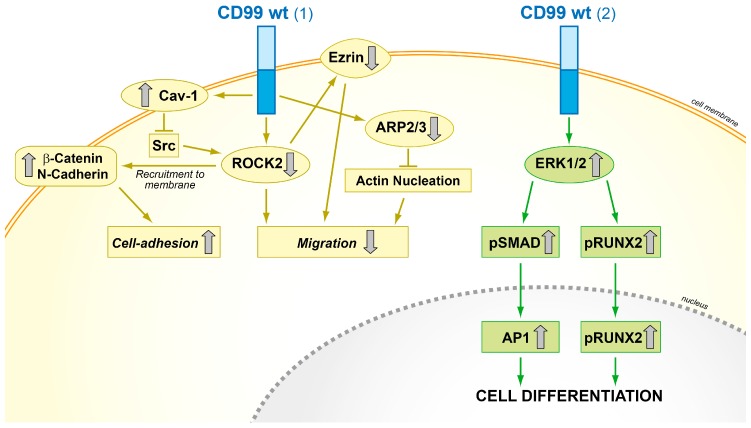
Schematic representation of the functional effects of forced CD99 expression in osteosarcoma cells. (1) CD99wt inhibits the c-Src/ROCK2 axis, which leads to ezrin inhibition, N-cadherin and β-catenin recruitment to the plasma membrane, and ARP2/3 downregulation, thereby increasing cell adhesion and reducing migration; (2) CD99wt upregulates ERK1/2 signaling and promotes the activity of the main osteogenic transcriptional factors AP1 and RUNX2, which in turn reactivate terminal differentiation [18,48,55].

From a mechanistic point of view, CD99 activity with respect to cell migration and invasion is at least partly related to the regulation of the cytoskeleton, actin remodeling, and increased expression of certain metalloproteinases, such as MMP-2 [15]. In osteosarcoma, transfection of CD99 inhibited tumor metastasis through the suppression of c-Src and Rho-associated, coiled-coil-containing protein kinase 2 (ROCK2) activities. CD99 forms stable complexes with caveolin-1, a molecule with oncosuppressor functions in osteosarcoma [86], and c-Src, which is therefore maintained in its inactive conformation [22]. Inhibition of c-Src functions is associated with decreased expression of ROCK2 and ezrin, a multifunctional protein that regulates cell adhesion and motility by connecting the actin cytoskeleton to the extracellular matrix [87]; by contrast, N-cadherin and β-catenin translocate to the plasma membrane and function as main molecular bridges for actin cytoskeleton. By favoring N-cadherin/β-catenin cell membrane recruitment, adherens junction formation and stable cell–cell interactions, the re-expression of CD99wt increases contact strength and reactivates stop-migration signals that counteract the otherwise dominant promigratory action of ezrin in osteosarcoma cells. In addition, the forced expression of CD99wt in osteosarcoma cells induced the downregulation of genes involved in actin cytoskeleton remodeling and cell invasion, such as actin-related protein 2 homologue (yeast) (ACTR2) and actin-related protein 2/3 complex, subunit 1A (ARPC1A) [55]; furthermore CD99 forced expression increased MAPK/ERK signaling, favoring the recruitment of activated ERK to the cell membrane/cytoplasm. The sustained activation of plasma membrane/cytoplasmic ERK in CD99-overexpressing cells may increase the activity of RUNX2 and BMP-SMAD-AP1 signaling, thus leading to increased activity of the master gene of osteoblastogenesis (Figure 5) [48].

In other tumors, however, the effect of CD99 on cytoskeletal organization and/or differentiation appears to be mediated by different molecular mechanisms, further supporting the strict cellular dependence of CD99 functions. In breast carcinoma MCF-7 cells, CD99 may function as a negative regulator of FAK-mediated tumorigenesis and metastasis by recruiting the protein tyrosine phosphatase SHP2 which dephosphorylates FAK and blocks the positive pro-tumorigenic signaling of CD98/integrins/FAK/RhoA/ROCK [88]. In Hodgkin’s lymphoma, CD99 induced the loss of multinucleated Reed–Sternberg cells morphology and redifferentiation [84] by regulating the expression of SEPTIN2, a member of the septin family that is involved in cell polarity and regulation of the actin and tubulin cytoskeleton (for a review see [89]), and STATHMIN, a cytoskeletal protein that is overexpressed in several malignancies and plays a significant role in cell differentiation [90], including B cell differentiation [91].

## 6. Therapeutic Perspectives of Strategies Targeting CD99

**Figure 6:**
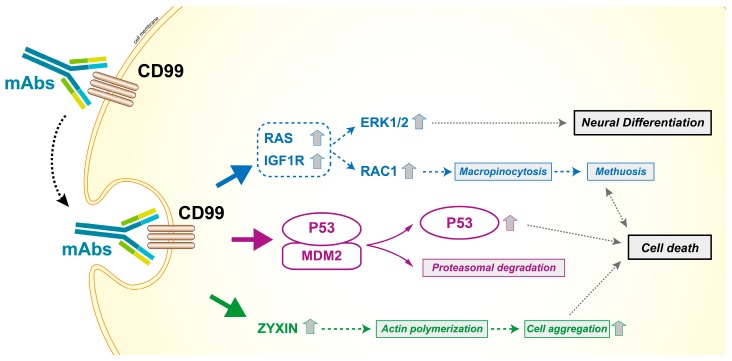
Effects of CD99 triggering by antibodies in Ewing sarcoma cells. CD99 engagement reduces the malignancy of tumor cells and induces caspase-independent cell death. The antibody-mediated engagement of CD99 induces a rapid caveolin-1-dependent endocytosis and promotes the upregulation of IGF1R and RAS signaling. The activation of the two downstream effectors Rac1 and ERK1/2 leads to macropinocytosis and death by methuosis or neural differentiation, respectively [59,96]. The effects are more dramatic in malignant cells that express high levels of CD99 and are facilitated by the reactivation of P53, resulting from the CD99-induced degradation of MDM2 [51,52,53,92]. Moreover, CD99 ligation upregulates zyxin that is associated with actin filaments and cell aggregation, a process also required for cell death [52].

As a cell surface molecule, CD99 can be easily targeted by antibodies [4,32,51,52,92,93]. The availability of a human bivalent antigen-binding antibody directed against CD99 (dAbd C7) [94,95] that is able to efficiently deliver a cell death message in Ewing sarcoma cells while sparing normal cells [53], opens new therapeutic perspectives for all tumors in which the expression of CD99 is maintained at high levels and supports tumor progression. CD99 engagement reduces the malignant potential of these tumors through different mechanisms, including the induction of caspase-independent programmed cell death [51,52,92] or methuosis [96], a process characterized by excessive accumulation of vacuoles in the cytoplasm, leading to compromised cell viability [97]. The capability to induce non-conventional apoptotic signaling may be clinically relevant, as tumor cells are generally resistant to classical apoptotic cell death. Accordingly, anti-CD99 antibodies exert additive/synergistic effects when combined with conventional agents, such as doxorubicin or vincristine [53,98], and are effective even against chemoresistant tumor cells [96]. Notably, the efficacy of CD99 antibodies is remarkably higher in, or exclusive to, cells carrying molecular alterations compared with normal hematopoietic or mesenchymal stem cells [4,32,53]; this finding further supports the therapeutic potential of anti-CD99 targeted therapies. Figure 6 summarizes the effects of CD99 triggering by antibodies. Most likely, tumor cells that are forced to grow by oncogenic stimuli cannot recover stressful signals such as those induced by CD99 triggering and thereby become more vulnerable than normal cells. Antibodies directed toward CD99 can also be used to develop radio–immuno compounds [99] that may detect CD99-positive tumors and metastatic sites with high sensitivity, outperforming FDG-PET in preclinical studies. This targeted radiotracer may have important implications for the diagnosis, surveillance, and treatment of tumors with high expression of CD99.

The selective expression of CD99 in immune and stromal cells [100] (for details, see the review [33]), in addition to representing a possible source of side effects for therapies targeting the molecule, may also constitute a possible source of therapeutic opportunities. CD99 ligation by antibodies can increase natural killer cell-mediated tumor lysis by inducing HSP70 expression [93]. In addition, peptides from CD99 have been described as promising candidates for immunotherapeutic glioblastoma treatment [101]. Branched multipeptides from ERBB2, BIRC5, and CD99 stably bound with T2 cells, and multipeptide-pulsed denditric cells–cytotoxic T lymphocytes exhibited remarkable cytotoxic activity against primary glioblastoma cells. Considering that peptide immunotherapy is affordable, easy to manufacture, and customizable to individual patients’ antigen profiles, this approach may offer interesting therapeutic perspectives. Notably, T cell receptor affinity-enhanced T cells have shown some efficacy in sarcomas [102]; in addition, hematological diseases and engineered transgenic, allo-restricted cytotoxic T cells have demonstrated in vitro and in vivo efficacy [103,104,105], rendering them a personalized treatment option for patients with an advanced disease.

Despite these potentialities, antibodies directed against CD99 have not reached the clinics yet. Recently, Çelik et al. described that the small molecule clofarabine was able to bind the extracellular portion of CD99, inhibiting the biological properties of Ewing sarcoma cells both in vitro and in vivo [106], thus suggesting a targeted use of an already developed drug.

CD99 has been recently demonstrated to be released in cell supernatants inside exosomes [74], which are small vesicles (40–130 nm) naturally produced by many cell types that entrap key elements necessary for intercellular communication. Exosomes are emerging as crucial messengers that can regulate physiological and pathological processes through the delivery of their content to recipient cells [107,108]. Notably, exosomes released by CD99-silenced Ewing sarcoma cells contain higher levels of miR34a than their parental counterparts and successfully downregulate Notch 1 and Notch 3 expression, as well as causing NF-κB transcriptional activity in recipient Ewing sarcoma cells, which in turn differentiate toward a neural phenotype [74].

This result opens new therapeutic avenues. In fact, exosome-mediated diffusion of the effects initiated by CD99 abrogation suggests that even a partial delivery of CD99 small interfering RNA (siRNA) may have a broad effect on an entire tumor cell population. On the other hand, it may be intriguing to explore the possibility of using exosomes to replace the effects of CD99 re-expression in tumors that have lost CD99 during progression.

Critical issues:

- In immune and tumor cells, CD99 is expressed in both short and long forms. Both isoforms play functional roles, and their differential expression can lead to distinct functional outcomes. Although the conjunction of the distinct functional outcomes via a single receptor might be coincidental, it is tempting to speculate that this finding represents a coordinated mechanism. The discrepant roles of CD99 in different tumors may be due not only to the difference in cancer types but also to the difference in the relative expression levels between the two CD99 isoforms. However, the currently available anti-CD99 antibodies cannot distinguish between the two CD99 isoforms, and other approaches must be considered in evaluating the expression levels of each CD99 isoform separately.
- The regulation of CD99 expression is still far from being understood. Considering the role that the loss of CD99 has in many common tumors, a true appreciation of the possible mechanisms involved (DNA methylation, LOH, transcriptional, and post-transcriptional mechanisms) is urgently required. In addition, more detailed studies are necessary to clarify how and when CD99sh is expressed with respect to the preeminent CD99wt isoform in different tumors.
- Considering the high dependence of CD99 functions on the cellular context, any therapeutic strategy targeting the molecule must be thoroughly studied in the appropriate context before any further clinical development.

New perspectives:

- The application of chimeric antigen receptor (CAR) T cell therapy is emerging as a very promising, novel strategy, particularly after its success in the treatment of certain hematologic cancers. However, CAR T cell therapy for solid tumors faces some challenges, and its application potential is still far from being fully exploited because of the lack of specific tumor antigens, tumor heterogeneity, and the presence of immunosuppressive factors in the tumor microenvironment [109,110]. CD99 and the recently developed dAbd C7 [53,94,95] may represent an opportunity to address the problem of enhancing the specificity of CAR T cells for those tumors with a maintained and diffuse expression of CD99 and might be considered for generating CARs that are capable of recognizing multiple antigens.
- Recent advances in our understanding of cancer have revealed that tumors should be considered as complex tissues in which cancer cells communicate directly and indirectly with the surrounding cellular microenvironment to promote their own survival [111,112]. Because it is expressed on specific immune and stromal cells in addition to normal and cancer stem cells, CD99 appears to be at the crossroad between the regulation of tumor cell malignancy and tumor interconnections with its microenvironment. A full appreciation of how the presence or absence of CD99 may affect tumor infiltration and the cross-talk between tumor and normal host cells may open new therapeutic avenues.
- Interactions between tumor cells and their microenvironment involve extracellular vesicles, which are released by cells under physiological and pathological conditions and contain a bioactive cargo (miRNA, mRNA, proteins and lipids). The presence of CD99 in exosomes may provide another perspective on the role of this molecule in tumorigenesis.

## 7. Concluding Remarks

CD99 is clearly involved in regulating tumor growth and differentiation and, even more importantly, in cell migration/adhesion and metastasis. In addition, the molecule is expressed in both immune and stromal cells and may have a function in the interplay between tumor cells and normal cells reacting to the tumor (e.g., mesenchymal stem cells, macrophages and T lymphocytes, and cancer-activated fibroblasts). However, the role of CD99 in tumorigenesis has been largely underestimated. The mechanisms of action are complex and strictly cell type-dependent, and a more profound level of understanding is required to fully exploit the therapeutic potential of strategies targeting CD99.
